# Agreement of Angiography-Derived and Wire-Based Fractional Flow Reserves in Percutaneous Coronary Intervention

**DOI:** 10.3389/fcvm.2021.654392

**Published:** 2021-04-23

**Authors:** Hu Ai, Naixin Zheng, Le Li, Guojian Yang, Hui Li, Guodong Tang, Qi Zhou, Huiping Zhang, Xue Yu, Feng Xu, Ying Zhao, Fucheng Sun

**Affiliations:** ^1^Department of Cardiology, National Center of Gerontology, Institute of Geriatric Medicine, Beijing Hospital, Chinese Academy of Medical Sciences, Beijing, China; ^2^The MOH Key Laboratory of Geriatrics, National Center of Gerontology, Beijing Hospital, Beijing, China

**Keywords:** fractional flow reserve, stable ischemic heart disease, percutaneous coronary intervention, vessel-oriented composite endpoint, coronary angiography-derived fractional flow reserve

## Abstract

**Background:** Coronary angiography-derived fractional flow reserve (caFFR) measurements have shown good correlations and agreement with invasive wire-based fractional flow reserve (FFR) measurements. However, few studies have examined the diagnostic performance of caFFR measurements before and after percutaneous coronary intervention (PCI). This study sought to compare the diagnostic performance of caFFR measurements against wire-based FFR measurements in patients before and after PCI.

**Methods:** Patients who underwent FFR-guided PCI were eligible for the acquisition of caFFR measurements. Offline caFFR measurements were performed by blinded hospital operators in a core laboratory. The primary endpoint was the vessel-oriented composite endpoint (VOCE), defined as a composite of vessel-related cardiovascular death, vessel-related myocardial infarction, and target vessel revascularization.

**Results:** A total of 105 pre-PCI caFFR measurements and 65 post-PCI caFFR measurements were compared against available wire-based FFR measurements. A strong linear correlation was found between wire-based FFR and caFFR measurements (*r* = 0.77; *p* < 0.001) before PCI, and caFFR measurements also showed a high correlation (*r* = 0.82; *p* < 0.001) with wire-based FFR measurements after PCI. A total of 6 VOCEs were observed in 61 patients during follow-up. Post-PCI FFR values (≤0.82) in the target vessel was the strongest predictor of VOCE [hazard ratio (HR): 5.59; 95% confidence interval (CI): 1.12–27.96; *p* = 0.036). Similarly, patients with low post-PCI caFFR values (≤0.83) showed an 8-fold higher risk of VOCE than those with high post-PCI caFFR values (>0.83; HR: 8.83; 95% CI: 1.46–53.44; *p* = 0.017).

**Conclusion:** The study showed that the caFFR measurements were well-correlated and in agreement with invasive wire-based FFR measurements before and after PCI. Similar to wire-based FFR measurements, post-PCI caFFR measurements can be used to identify patients with a higher risk for adverse events associated with PCI.

## Introduction

Angiography-derived fractional flow reserve (FFR) measurements represent a novel technique for evaluating physiological function in cardiovascular disease (1, 2). Over the past few years, four angiography-derived FFR measurement methods have shown good correlation and agreement with the conventional invasive wire-based FFR method in the FAVOR II China study (3), FAST-FFR study (4), FLASH FFR study (5), and FAST study (6). Among patients who are suspected of coronary heart disease, these clinical trials have shown that angiography-derived FFR measurement techniques have good diagnostic performance for guiding revascularization in percutaneous coronary intervention (PCI). However, few studies have examined the diagnostic performance of coronary angiography-derived FFR (caFFR) before and after PCI. The value of initial wire-derived FFR is typically below 0.8 among patients who have previously undergone PCI, which might challenge the diagnostic abilities of caFFR. Furthermore, whether computational fluid dynamics have diagnostic value in coronary arteries implanted with exogenous metal stents remains unknown.

The objective of the current retrospective study was to compare the diagnostic performance of caFFR measurement against wire-based FFR measurement among patients before and after PCI. The FlashAngio caFFR system includes the Flash pressure transducer, console, and software (Rainmed Ltd., Suzhou, China). In this study, pre-PCI and post-PCI caFFR measurements were compared with corresponding wire-based FFR measurements. We also investigated the post-PCI FFR and caFFR cutoff values for the prediction of long-term adverse cardiac outcomes.

## Methods

### Study Population

Patients (≥18 years of age) with stable ischemic heart disease (SIHD) who underwent elective invasive FFR-guided PCI for a *de novo* lesion from June 2012 to May 2020 at Beijing Hospital were included in this study. The angiographic inclusion criterion was at least one lesion with diameter stenosis of 50–90% by visual assessment. The angiographic exclusion criteria (5), as required by the FlashAngio caFFR system, included: (1) poor angiographic image quality, precluding contour detection; (2) severe vascular overlap or distortion of the interrogated vessel; (3) stenoses caused by myocardial bridge; and (4) ostial lesions. The clinical data were obtained from electronic medical records and analyzed retrospectively. The study was approved by the Institutional Ethics Committee (2020BJYYEC-038-01) at Beijing Hospital. All patients signed informed consent to undergo invasive FFR-guided PCI and agreed to the use of their data for research purposes.

### Determination of Wire-Based FFR

Intracoronary nitroglycerine (200 mg) was routinely injected before FFR measurement. The coronary pressure wire-based FFR was measured using a commercially available pressure wire system (Certus, Abbott Vascular, Santa Clara, CA). The pressure wire was inserted such that the pressure transducer was ≥2 cm downstream from the most distal stenosis. The position of the pressure wire was captured on cine angiography for offline comparisons. Hyperemic blood flow was induced by the intravenous administration of adenosine-5'-triphosphate (ATP) at ≥140 μg/kg/min and recorded after at least 60 s in the presence of stable aortic pressure decrease relative to baseline pressure that was sustained for at least 10 beats. FFR pullback was performed at the operator's judgment. Pressure drift was measured after the withdrawal of the pressure wire to the guiding catheter tip and was defined as a resting distal-to-aortic coronary pressure ratio (Pd/Pa) from 0.97 to 1.03.

### Coronary Revascularization and Image Transfer

Coronary angiography was performed based on 9 conventional projection views (7), which were recorded at 15 frames/s. A mechanical pump was used to inject the contrast agent at a rate of 3.5 mL/s. The PCI procedures were determined by an interventional cardiologist following the best local practices. At the end of the procedure, at least two angiographic projection views for the targeted vessel were recorded. During the operation, the aortic pressure value was routinely recorded in the Catheter Laboratory Database.

### Offline caFFR Measurement

**Figure 1:**
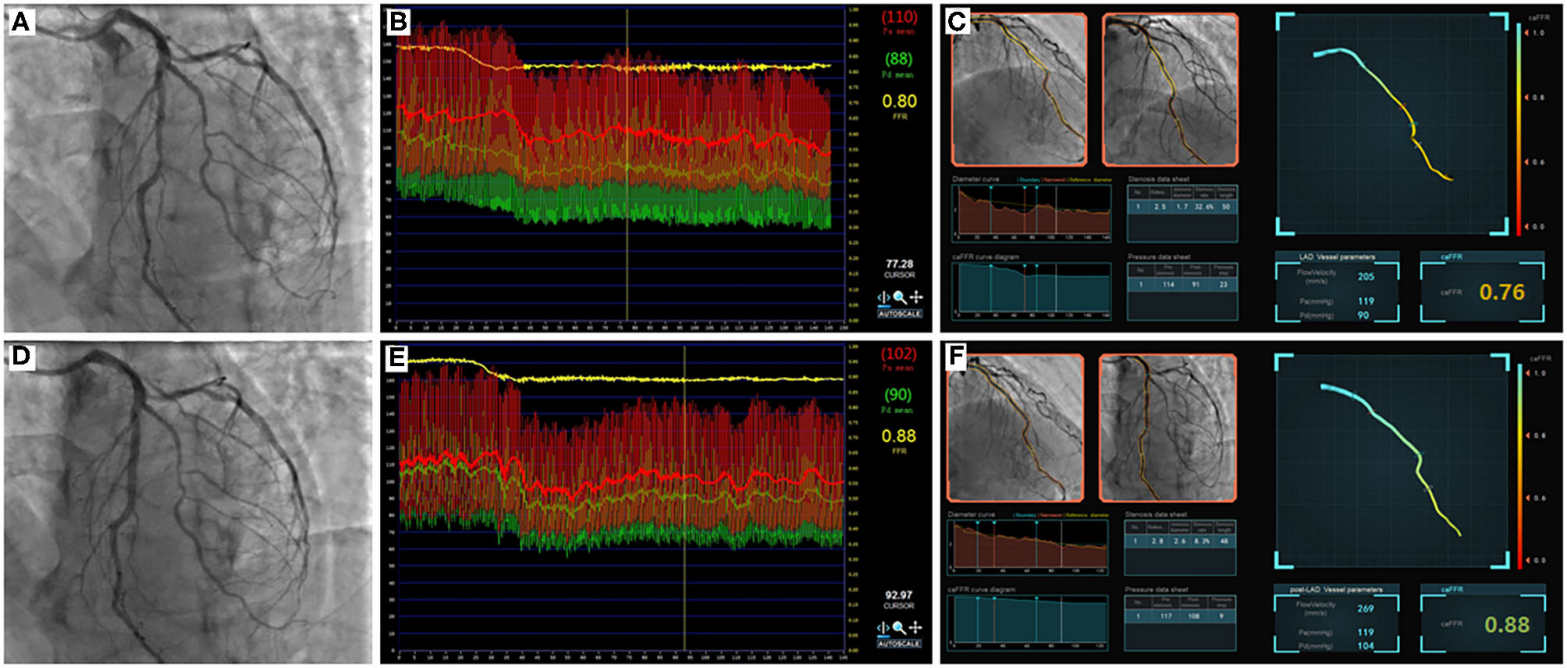
Example of comparisons between pre- and post-PCI FFR and caFFR values. Images were obtained from a 61-year-old patient in the study. **(A)** Coronary angiography shows moderate stenosis lesion located in the middle of the LAD. **(B)** The value of pre-PCI FFR, measured by invasive pressure wire, was 0.80. **(C)** The pre-PCI caFFR value was 0.76. **(D)** Post-PCI coronary angiography after 2.5 × 24 mm and 2.75 × 24 mm stent implantation. **(E)** The invasive post-PCI wire-based FFR value was 0.88. **(F)** The post-PCI caFFR value was 0.88. LAD, left descending artery; FFR, fractional flow reserve; PCI, percutaneous coronary intervention; caFFR, coronary angiography-derived FFR.

At least two angiographic projections, avoiding vessel overlap and separated by ≥30°, without table movement, were required to generate caFFR. Digital Imaging and Communications in Medicine (DICOM) images of coronary angiography and mean aortic pressure (MAP) were exported to the FlashAngio console. A simulated three-dimensional (3D) mesh reconstruction of the coronary artery was generated along the artery path from the inlet to the most distal location. The caFFR computation was performed by blinded hospital operators using the method described previously (5). The values of pre-PCI and post-PCI caFFR were reported separately, as shown in Figure 1.

### Follow-Up and End Points

Clinical follow-up data were recorded in a dedicated database, including admission records and outpatient notes, maintained at Beijing Hospital. All patients were followed individually by direct telephone contact or outpatient visits to confirm clinical data every 6 months. The primary endpoint was the vessel-oriented composite endpoint (VOCE), defined as the composite of vessel-related cardiovascular death, vessel-related myocardial infarction (MI), and target vessel revascularization (TVR) (8). Secondary endpoints were the individual components of the VOCE. Death of unknown etiology was considered cardiovascular death. MI was defined as new Q waves or one plasma level of creatine kinase-myocardial band (CK-MB) ≥ 5 × upper limit of normal (ULN; or troponin ≥ 35 × ULN if CK-MB was not available) in the context of acute coronary syndrome (ACS) (9). TVR was defined as the repeat revascularization or bypass grafting of the target vessel.

### Statistical Analysis

Continuous variables are expressed as the mean and standard deviation. Categorical data are summarized as the number and percentage. The Student's *t*-test and the Chi-square test were used to compare group differences. The correlation between wire-based FFR and caFFR measurements was assessed by Spearman's correlation coefficient (r) with a 95% confidence interval (CI). Bland–Altman analysis was used to estimate the agreement between the two indices. Patients were separated into two groups (high-risk and low-risk groups) according to the post-PCI wire-based FFR and caFFR values. The cumulative survival probability of VOCE was estimated by Kaplan–Meier curves; the difference between high-risk and low-risk groups was compared by a log-rank test and plotted using the “survival” package of R language (Version 3.6.1). In parallel, Cox regression was fitted to estimate the risks of VOCE (hazard ratio [HR], 95% CI) for the two groups. The cutoff post-PCI FFR value for the prediction of long-term adverse cardiac outcomes varied from 0.81 to 0.95 (10). We tested the threshold starting at 0.81 to determine the optimal cutoff value for post-PCI FFR measurements to predict VOCE to determine whether caFFR measurements have similar prognostic power as traditional wire-based FFR measurements for VOCE prediction. All statistical analyses were performed with SPSS software (Version 24.0, IBM Corp., Armonk, NY) and R language. The significance level was set at *p* < 0.05, and all probability values were two-sided.

## Results

### Feasibility and Characteristics of Patients

**Figure 2:**
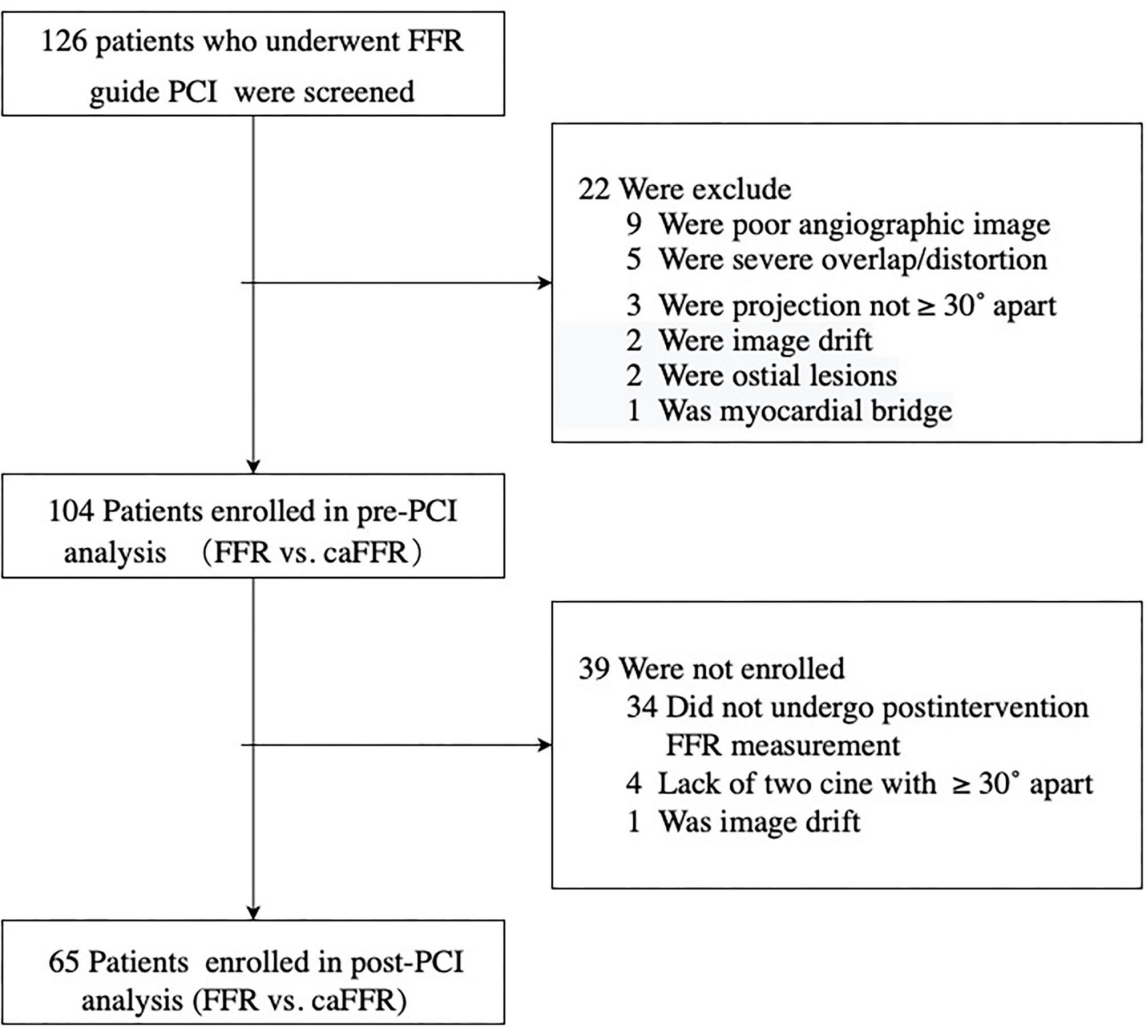
Study flow diagram. SIHD, stable ischemic heart disease; LAD, left descending artery; FFR, fractional flow reserve; PCI, percutaneous coronary intervention; caFFR, coronary angiography-derived FFR.

**Figure 3:**
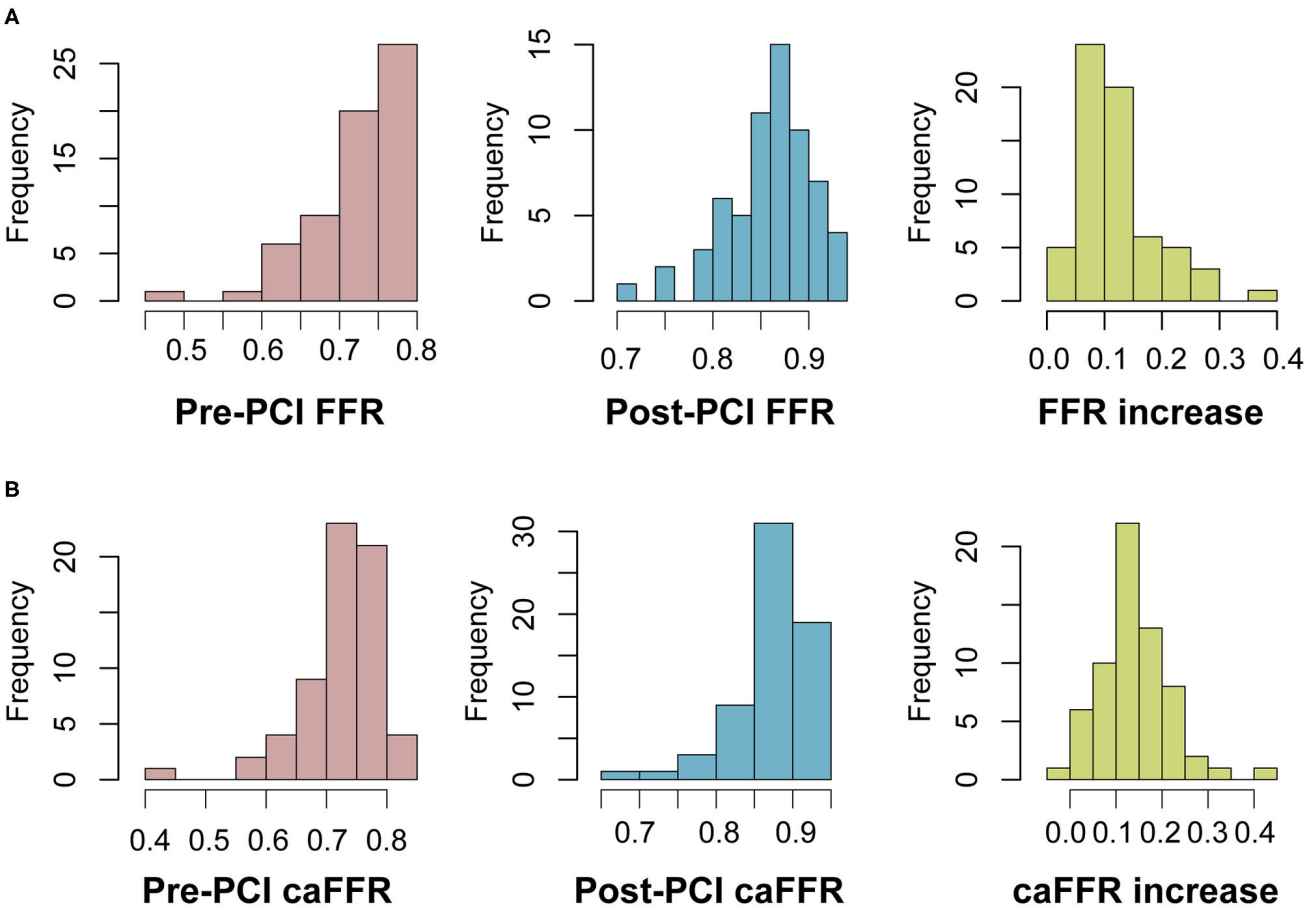
Frequency distribution of FFR and caFFR values and increased values. **(A)** Frequency distribution and increased values of FFR. **(B)** Frequency distribution and increased values of caFFR. LAD, left descending artery; FFR, fractional flow reserve; PCI, percutaneous coronary intervention; caFFR, coronary angiography-derived FFR.

From 2012 to 2020, 126 patients with SIHD who underwent FFR-guided PCI were enrolled in this study, and caFFR was analyzed in 104 patients (105 vessels). Post-PCI FFR measurement was performed in 70 patients (67.3%, 71 vessels), and post-PCI caFFR measurements could be performed in 65 patients (65 vessels). The primary causes of caFFR computation failure were poor image quality (*n* = 9 of 126, 7.14%), severe overlap or distortion (*n* = 5 of 126, 3.97%), and the lack of 2 cines with projection angles ≥ 30° (*n* = 3 of 126, 2.38%), as shown in Figure 2. Figure 3 shows the frequency distribution of FFR measurements, caFFR measurements, and increased relative values to baseline. Clinical and interventional characteristics are reported in Table 1.

**Table 1:** Clinical and angiographic characteristics of patients.

**Patients (*n* = 104)**	
Sex, male (%)	78 (75.0)
Age (years)	61.6 ± 9.6
BMI (kg/m^2^)	25.9 ± 3.5
Smoking (%)	43 (41.3)
Hypertension (%)	67 (64.4)
Diabetes (%)	42 (40.4)
Dyslipidemia (%)	69 (66.3)
Hemoglobin (g/L)	135.0 ±14.8
FBG (mmol/L)	6.4 ± 2.0
Creatinine (μmol/L)	71.6 ± 17.2
LDL-C (mmol/L)	2.1 ± 0.8
LVEF (%)	64.2 ± 5.4
Multivessel disease, *n* (%)	55 (52.9)
**Target vessel (*n* = 105)**	
**Location of lesion**	
LAD, *n* (%)	96 (91.4)
LCX, *n* (%)	5 (4.8)
RCA, *n* (%)	4 (3.8)
**ACC/AHA lesion type**	
A (%)	17 (16.2)
B_1_ (%)	29 (27.6)
B_2_ (%)	25 (23.8)
C (%)	34 (32.4)
**Quantitative coronary angiography**	
**Pre-PCI**	1.6 ± 0.4
Minimal lumen diameter, mm	
Reference vessel diameter, mm	3.0 ± 0.6
% Diameter stenosis	48.5 ± 9.3
Lesion length, mm	19.3 ± 10.0
**Post-PCI**	
Minimal lumen diameter, mm	2.4 ± 0.5
Reference vessel diameter, mm	2.8 ± 0.5
% Diameter stenosis	14.3 ± 7.1
Lesion length, mm	14.7 ± 8.5
**Interventional characteristics**	
DES implantation	80 (76.19)
DCB dilation	25 (23.81)

*FFR, fractional flow reserve; caFFR, coronary angiography-derived FFR; BMI, body mass index; FBG, fasting blood glucose; LDL-C, low-density lipoprotein cholesterol; LVEF, Left ventricular ejection fraction; LAD, left anterior descending artery; RCA, right coronary artery; LCX, left circumflex artery; ACC, American College of Cardiology; AHA, American Heart Association; PCI, percutaneous coronary intervention; DES, drug-eluting stent; DCB, drug-coated balloon*.

### Agreement Between FFR and caFFR

**Figure 4:**
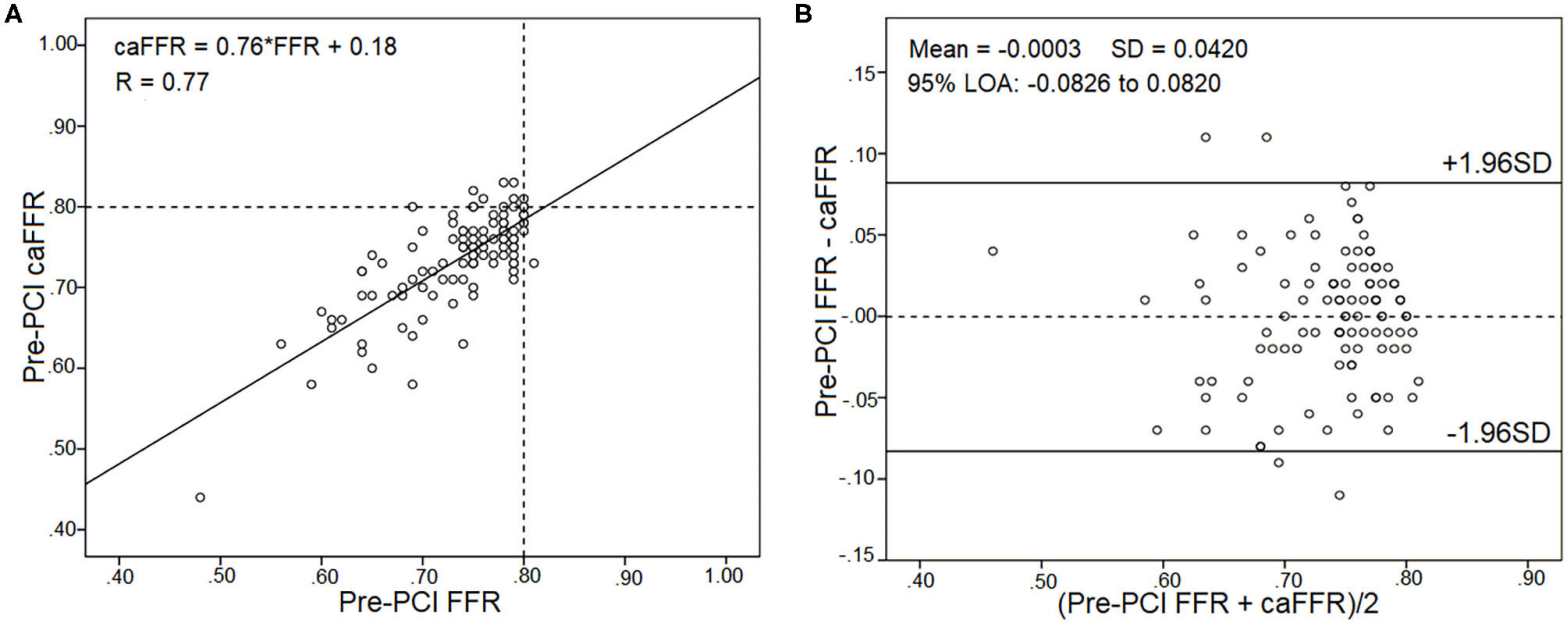
Correlation and agreement between pre-PCI wire-based FFR and caFFR values (*n* = 105). **(A)** Strong correlation between pre-PCI wire-based FFR and caFFR values (caFFR = 0.76 × FFR + 0.18, *R* = 0.77, 95% CI: 0.53–0.78). **(B)** Good agreement between pre-PCI wire-based FFR and caFFR values by the Bland–Altman analysis (bias: −0.0003 ± 0.0420; 95% LOA −0.0826 to 0.0820). CI, confidence interval; LOA, limits of agreement; LAD, left descending artery; FFR, fractional flow reserve; PCI, percutaneous coronary intervention; caFFR, coronary angiography-derived FFR.

**Figure 5:**
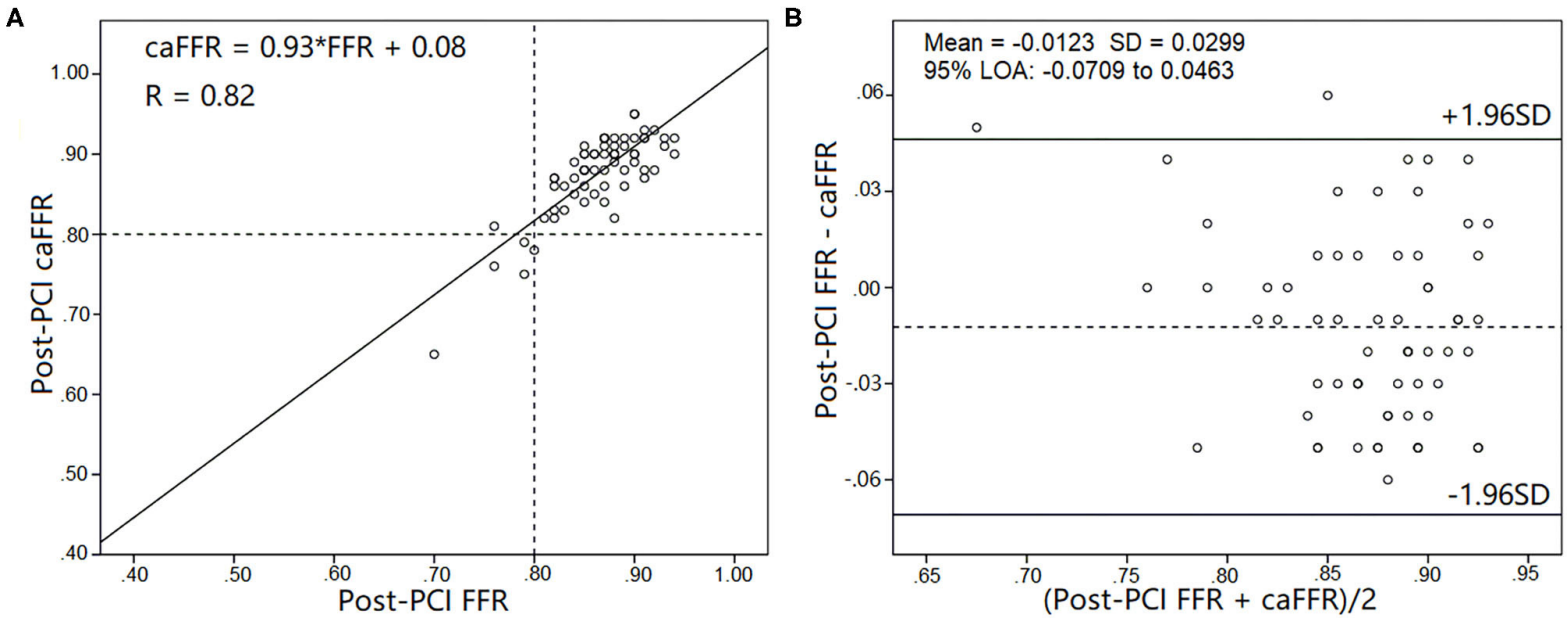
Correlation and agreement between post-PCI wire-based FFR and caFFR values (*n* = 65). **(A)** Strong correlation between post-PCI wire-based FFR and caFFR values (caFFR = 0.93 × FFR + 0.08, *R* = 0.82, 95% CI: 0.762–1.092). **(B)** Good agreement between post-PCI wire-based FFR and caFFR values by the Bland–Altman analysis (bias: −0.0123 ± 0.0299; 95% LOA −0.0709 to 0.0463). CI, confidence interval; LOA, limits of agreement; LAD, left descending artery; FFR, fractional flow reserve; PCI, percutaneous coronary intervention; caFFR, coronary angiography-derived FFR.

**Figure 6:**
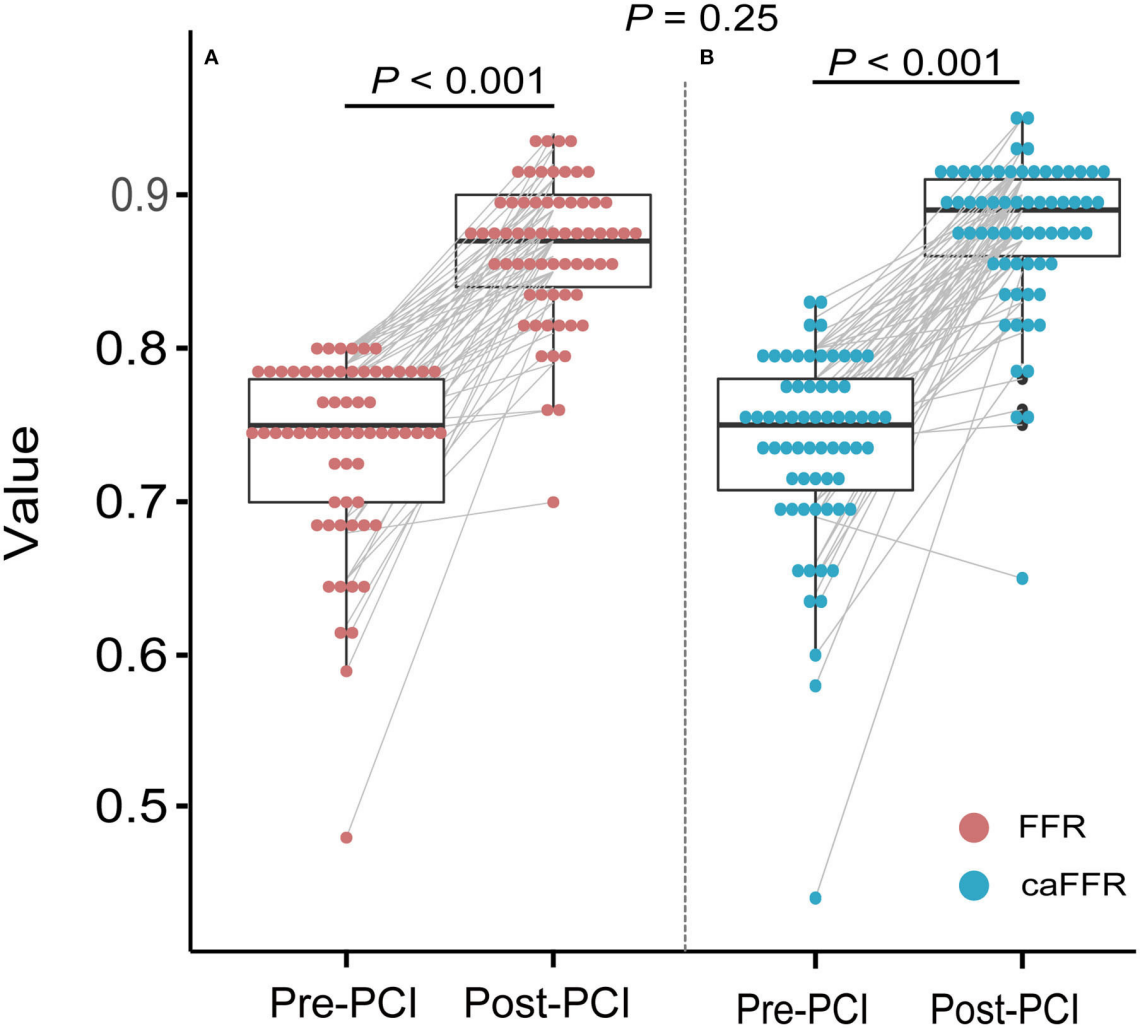
Improvements in wire-based FFR and caFFR values after PCI (*n* = 65). **(A)** The wire-based FFR values increased significantly after PCI (0.74 ± 0.07 vs. 0.86 ± 0.05, *p* < 0.001). **(B)** The caFFR values increased significantly after PCI (0.74 ± 0.07 vs. 0.88 ± 0.05, *p* < 0.001). The increase in wire-based FFR values after PCI were similar to that of caFFR values (0.13 ± 0.07 vs. 0.14 ± 0.08; *p* = 0.25). LAD, left descending artery; FFR, fractional flow reserve; PCI, percutaneous coronary intervention; caFFR, coronary angiography-derived FFR.

Pre-PCI caFFR values were well-correlated with wire-based FFR values (caFFR = 0.76 × FFR + 0.18, *R* = 0.77, Figure 4A). The Bland–Altman analysis of pre-PCI caFFR and wire-based FFR values showed no systematic differences, with a bias of −0.0003 ± 0.0420 (95% limit of agreement: −0.0826 to 0.0820, Figure 4B). The post-PCI caFFR values were also correlated with wire-based FFR values (caFFR = 0.93 × FFR + 0.88, *R* = 0.817, Figure 5A). Bland–Altman analysis of post-PCI caFFR and wire-based FFR values showed no systematic differences, with a bias of −0.0123 ± 0.0299 (95% limit of agreement: −0.0709 to 0.0463, Figure 5B). Figure 6 shows the increase in the wire-based FFR values after PCI, similar to those observed by caFFR measurement (0.13 ± 0.07 vs. 0.14 ± 0.08; *p* = 0.25).

### Prognostic Implications of Post-PCI caFFR

**Figure 7:**
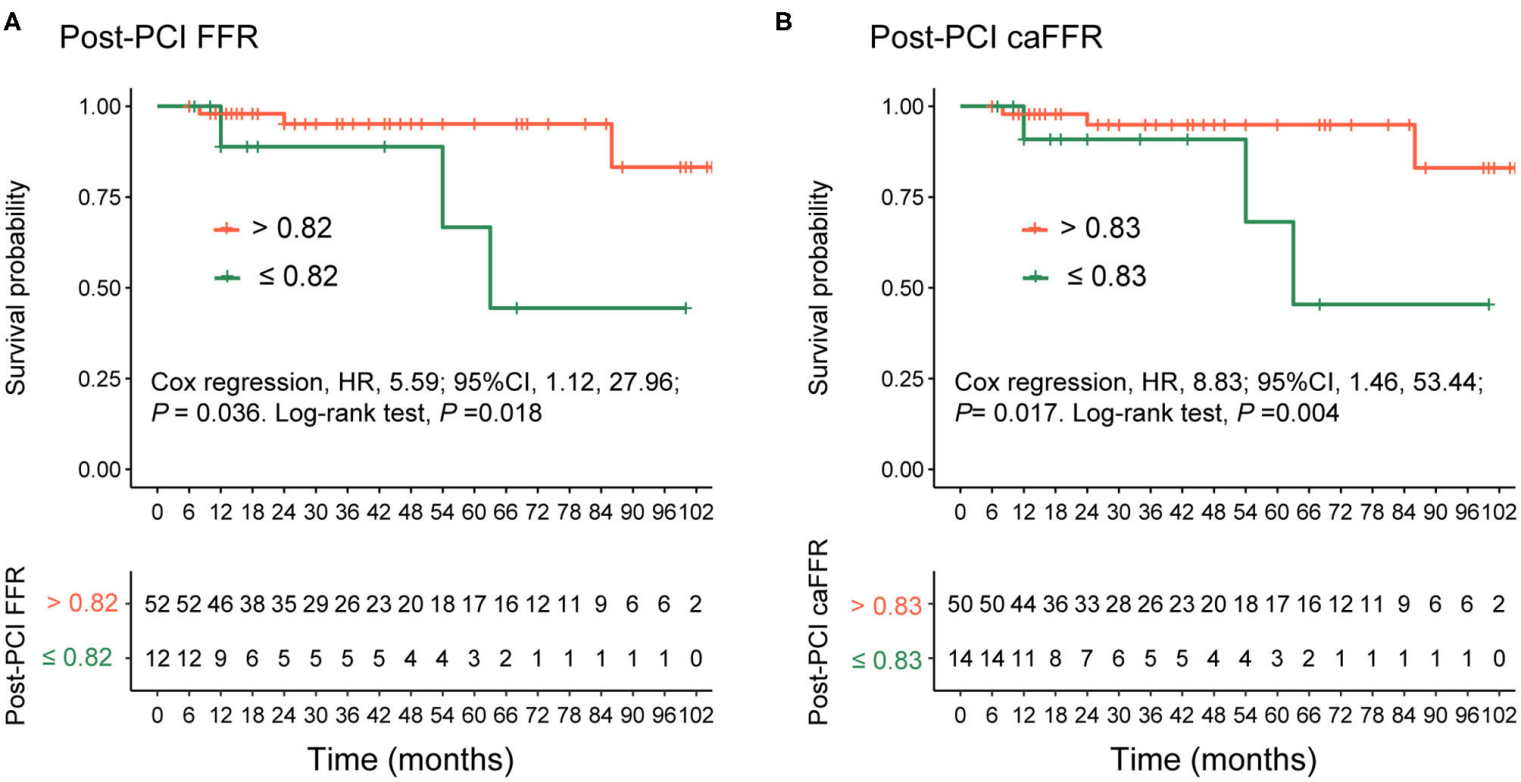
Comparison of VOCEs according to post-PCI FFR or caFFR values. Kaplan–Meier curves showing that **(A)** patients with post-PCI wire-based FFR values > 0.82 had a higher survival free of VOCE compared with patients with post-PCI wire-based FFR values ≤ 0.82. **(B)** Patients with post-PCI caFFR values > 0.83 had a higher survival free of VOCE compared with patients with post-PCI caFFR values ≤ 0.83 group. VOCE, vessel-oriented composite endpoint; LAD, left descending artery; FFR, fractional flow reserve; PCI, percutaneous coronary intervention; caFFR, coronary angiography-derived FFR.

In 65 patients with post-PCI wire-based FFR and caFFR measurements, 61 (93.9%) patients had clinical follow-up data. During a follow-up period of 6–105 months (86.9% >12-month follow-up; 50.9% >36-month follow-up), 6 (9.8%) VOCEs were repo rted: 1 cardiovascular death (1.6%), 1 MI (1.6%), and 4 TVRs (6.6%). The post-PCI caFFR values showed consistent power with post-PCI wire-based FFR values for the prediction of VOCEs, although different cutoff values were identified for the two measurement methods (Supplementary Figure 1). Patients with low post-PCI wire-based FFR values (≤ 0.82) had a higher risk of VOCE than those with high post-PCI wire-based FFR values (> 0.82; HR: 5.59; 95% CI: 1.12–27.96; *p* = 0.036). Similarly, patients with low post-PCI caFFR values (≤ 0.83) showed an 8-fold higher risk of VOCE than those with high post-PCI caFFR values (> 0.83; HR: 8.83; 95% CI: 1.46–53.44; *p* = 0.017; Figure 7).

When the patients were grouped according to their post-PCI wire-based FFR values (>0.82 vs. ≤ 0.82; Table 2), a significant difference was found in the numbers and total lengths of the stents used (1.50 ± 0.55 vs. 1.0 and 30.96 ± 10.45 vs. 19.75 ± 3.73, respectively; *p* < 0.01). Similarly, both the numbers and total lengths of stents in the high caFFR value group (>0.83 vs. ≤ 0.83) were significantly higher than those in the low caFFR value group (1.48 ± 0.55 vs. 1.0; 30.52 ± 10.51 vs. 19.50 ± 2.66; *p* < 0.01; Table 2).

**Table 2:** Comparison of general and procedural characteristics according to post-PCI wire-based FFR and caFFR values.

	**Post-PCI wire-based FFR value**			**Post-PCI caFFR value**		
	**High FFR (>0.82, *n* = 53)**	**Low FFR (≤0.82, *n* = 12)**	***P*-Value**	**High caFFR (>0.83, *n* = 54)**	**Low caFFR (≤0.83, *n* = 11)**	***P*-Value**
Sex, male (%)	41 (77.36)	10 (83.33)	0.948	43 (79.63)	8 (72.73)	0.916
Age (years)	61.21 ± 9.97	59.75 ± 9.98	0.649	60.89 ± 9.73	61.18 ± 11.25	0.93
BMI (kg/m^2^)	26.02 ± 3.37	27.74 ± 6.26	0.351	25.84 ± 3.23	28.22 ± 6.57	0.07
Smoking (%)	22 (41.51)	8 (66.67)	0.208	25 (46.30)	5 (45.45)	1.0
Hypertension (%)	33 (63.26)	7 (58.33)	0.801	33 (61.11)	7 (63.64)	0.875
Diabetes (%)	19 (35.85)	4 (33.33)	0.869	19 (35.19)	4 (36.36)	0.941
Dyslipidemia (%)	34 (64.15)	10 (83.33)	0.347	36 (66.67)	8 (72.73)	0.970
**Target lesion location**						
LAD, *n* (%)	50 (94.34)	11 (91.67)	0.567	51 (94.44)	10 (90.91)	0.533
LCX, *n* (%)	1 (1.89)	1 (8.33)		2 (3.70)	0	
RCA, *n* (%)	2 (3.77)	0		1 (1.85)	1 (9.09)	
**ACC/AHA lesion type**						
A (%)	8 (0.15)	1 (8.33)	0.889	8 (14.81)	1 (9.09)	0.296
B_1_ (%)	13 (24.53)	3 (25.00)		13 (24.07)	3 (27.27)	
B_2_ (%)	12 (22.64)	4 (33.33)		11 (20.37)	5 (45.45)	
C (%)	20 (37.74)	4 (33.33)		22 (40.74)	2 (18.18)	
**Quantitative coronary angiography**						
Pre-PCI reference vessel diameter, mm	3.05 ± 0.62	2.96 ± 0.49	0.699	3.05 ± 0.63	2.98 ± 0.39	0.676
Pre-PCI diameter stenosis, %	49.24 ± 8.95	44.68 ± 5.32	0.028	49.38 ± 8.89	43.57 ± 4.36	0.039
Pre-PCI lesion length, mm	19.96 ± 10.29	17.18 ± 8.47	0.389	20.22 ± 10.33	15.61 ± 7.28	0.093
Post-PCI diameter stenosis, %	12.71 ± 6.31	13.54 ± 3.41	0.662	12.90 ± 6.08	12.70 ± 4.95	0.917
**Procedural characteristics**						
DES/DCB	46/7	8/4	0.21	48/6	6/5	0.020
Number of DES	1.50 ± 0.55	1.00	0.000	1.48 ± 0.55	1.00	0.000
Diameter of DES, mm	3.08 ± 0.46	2.75 ± 0.34	0.08	3.08 ± 0.46	2.70 ± 0.25	0.06
Total length of DES, mm	30.96 ± 10.45	19.75 ± 3.73	< 0.001	30.52 ± 10.51	19.50 ± 2.66	< 0.001
Number of DCB	1.14 ± 0.38	1.50 ± 0.58	0.241	1.17 ± 0.41	1.40 ± 0.51	0.438
Diameter of DCB, mm	3.14 ± 0.28	3.00 ± 0.35	0.479	3.17 ± 0.30	3.00 ± 0.31	0.389
Total length of DCB, mm	20.71 ± 6.73	28.75 ± 9.46	0.132	20.83 ± 7.35	27.00 ± 9.08	0.244

*FFR, fractional flow reserve; caFFR, coronary angiography-derived FFR; BMI, body mass index; FBG, fasting blood glucose; LDL-C, low-density lipoprotein cholesterol; LVEF, Left ventricular ejection fraction; LAD, left anterior descending artery; RCA, right coronary artery; LCX, left circumflex artery; ACC, American College of Cardiology; AHA, American Heart Association; PCI, percutaneous coronary intervention; DES, drug-eluting stent; DCB, drug-coated balloon*.

## Discussion

The present study validated caFFR measurement against those obtained using conventional wire-based FFR measurements before and after PCI. The major findings were as follows: (1) caFFR analysis could be applied to most conventional coronary angiography images; (2) caFFR and wire-based FFR measurements had good correlation and agreement both before and after PCI; and (3) post-PCI wire-based FFR and caFFR measurements had similar prognostic values.

FFR measurement has been used in the cardiac catheter laboratory to identify functionally significant coronary stenoses (11, 12). Random trials have demonstrated that FFR-guided PCI improves clinical outcomes and reduces the need for stenting, in addition to reducing costs (13–15). FFR measurement is recommended as a standard protocol in the revascularization guidelines for stable coronary heart disease (16, 17). Angiography-derived FFR and wire-based FFR measurements showed similar diagnostic accuracy. Using wire-based FFR as the reference standard, the area under the curve (AUC) for caFFR (5) was 0.979, and similar results were demonstrated in studies of the quantitative flow ratio (QFR) (3), FFRangio (4), and Vessel FFR (vFFR) (6) measurement methods, which reported AUC values of 0.96, 0.94, and 0.93, respectively. In this study, pre-PCI caFFR values showed good diagnostic accuracy in patients who underwent elective PCI for a *de novo* lesion compared with the diagnostic accuracy of wire-based FFR values.

FFR measurement after PCI can be used to discriminate suboptimal PCI procedures and predict clinical outcomes. Pijls et al. (18) investigated 750 patients following successful bare-metal stent implantation and found that lower post-PCI FFR values were associated with an increased major adverse cardiovascular event (MACE) rate (for groups based on FFR values > 0.95, 0.8–0.9, and < 0.80, MACE rates were 4.9% 20.3% and 29.5%, respectively; *p* < 0.05) at the 6-month follow-up time point. The DK CRUSH VII Registry Study (19) revealed that a post-PCI FFR value < 0.88 was the predominant predictor of target vessel failure (12.3 vs. 6.1%; *p* < 0.01) 3 years after drug-eluting stent implantation. An increase in the FFR value after PCI was directly associated with MI recovery (20), whereas a small increase in the FFR value (<15%) was a prognostic indicator of poor clinical outcomes, similar to low absolute post-PCI FFR values (<0.84) (21). The FFR-search study unexpectedly showed that post-PCI FFR values did not correlate with clinical events at the 30-day follow-up time point (22), which was thought to be due to the follow-up period being too short. The reasons for ineffective FFR changes after PCI include incomplete stent expansion, stent malapposition, geographical plaque miss, plaque protrusion, edge dissection, and plaque shift at the stent edge (23). To achieve functional optimization, unsatisfactory changes in FFR can safely and effectively be corrected by further interventions (post-dilation or additional stenting) (24).

Despite a Class 1a recommendation for use in the guidance of coronary revascularization in patients with stable angina, only 18.5%−21% of patients undergo FFR measurement (25, 26). Moreover, post-PCI FFR measurement was only performed in 69.2 and 64.2% of patients in the FAME 1 and FAME 2 studies (27). caFFR measurement represents a new technique that can be performed without the use of a pressure wire and hyperemic stimulus. The FLASH FFR study demonstrated that caFFR measurement has a good correlation with wire-based FFR measurement, and caFFR measurement requires a shorter operation time (< 5 min) than wire-based FFR measurement (5). The present study demonstrated that caFFR measurement had a good correlation with wire-based FFR measurement both before and after PCI. Similarly, post-PCI QFR and vFFR measurements correlated reasonably well-with post-PCI wire-based FFR measurements (28, 29). However, in a retrospective study of data collected by more than 50 centers, a weak correlation was reported between vFFR measurement and wire-based FFR measurement because of poor image quality and the difficulty tracking aortic pressure (30). The prognostic value of QFR measurements has been confirmed under various conditions (8, 9, 31), although the optimal cutoff values for post-PCI QFR measurements have been reported as 0.89, 0.91, and 0.80 in different studies. In the current single-center, retrospective study, patients with low post-PCI wire-based FFR (≤0.82) or caFFR (≤0.83) values were associated with a significantly higher risk of VOCE than those with high post-PCI FFR (>0.82) or caFFR (>0.83) values. The numbers and lengths of stents implanted in the high post-PCI FFR and caFFR value groups were significantly higher than those in the low post-PCI FFR and caFFR value groups. Insufficient stent implantation has been shown to result in incomplete coronary lesion coverage, which may explain the suboptimal FFR and caFFR values measured after the intervention in some patients. Currently, no consensus exists regarding the optimal post-PCI FFR value, although a higher FFR value is generally believed to be preferable. This study showed that a higher post-PCI caFFR value could predict a better clinical outcome.

## Limitations

The present study has several limitations. First, this study was performed as a retrospective study in a single center with a relatively small study population. Second, only patients with stable coronary heart disease were enrolled in this study. High-risk patients, such as those with ACS, ostial lesions, or left main lesions, were excluded, which restrains the generalizability of the results. Third, aortic root pressure was obtained from an interventional database maintained by the cardiac catheterization laboratory rather than measuring real-time invasive pressure. The retrospective methodology of obtaining aortic root pressure might affect the results slightly. Fourth, the retrospective study lacked a sufficient sample size and preconditions. We cannot recommend a powerful post-PCI FFR or caFFR cutoff value for the prediction of long-term adverse cardiac outcomes, and we did not obtain sufficient adverse event data to analyze any other VOCE predictors. Finally, macrovascular and microvascular diseases can affect coronary physiology after PCI. Evidence suggests that microvascular dysfunctions may falsify the results of FFR and be associated with adverse events after PCI (32–34). We will perform experimental and computational analyses of post-PCI microvascular dysfunction in future studies to quantify this effect.

## Conclusions

This study showed that caFFR measurements are feasible, reproducible, and well-correlated with invasive wire-based FFR measurements both before and after PCI. Similar to wire-based FFR measurements, post-PCI caFFR measurements might be a useful tool for performing coronary physiological functional assessments and identifying patients with a higher risk for adverse events related to PCI.

## Data Availability Statement

The original contributions presented in the study are included in the article/Supplementary Material, further inquiries can be directed to the corresponding authors.

## Ethics Statement

The studies involving human participants were reviewed and approved by The institutional Ethics Committee approved the study at Beijing Hospital (decision no. 2020BJYYEC-038-01). The patients/participants provided their written informed consent to participate in this study. Written informed consent was obtained from the individual(s) for the publication of any potentially identifiable images or data included in this article.

## Author Contributions

HA, NZ, and LL: study conception and design. NZ, LL, GY, HL, GT, HZ, XY, and FX: acquisition of data. HA and QZ: analysis and interpretation of data. HA: writing, review, and/or revision of the manuscript. YZ and FS: study supervision. All authors contributed to the article and approved the submitted version.

## Conflict of Interest

The authors declare that the research was conducted in the absence of any commercial or financial relationships that could be construed as a potential conflict of interest.

## Supplementary Material

Supplementary Figure 1 Subjects whose post-PCI wire-based FFR or caFFR values were lower than the cutoff values were categorized as the high-risk groups. LAD, left descending artery; FFR, fractional flow reserve; PCI, percutaneous coronary intervention; caFFR, coronary angiography-derived FFR.
