# Supplementary figures and images for: Agreement of Angiography-Derived and Wire-Based Fractional Flow Reserves in Percutaneous Coronary Intervention
Source: Front Cardiovasc Med. 2021 Apr 23;8:654392. doi: 10.3389/fcvm.2021.654392 (PMC8102686; doi:10.3389/fcvm.2021.654392)

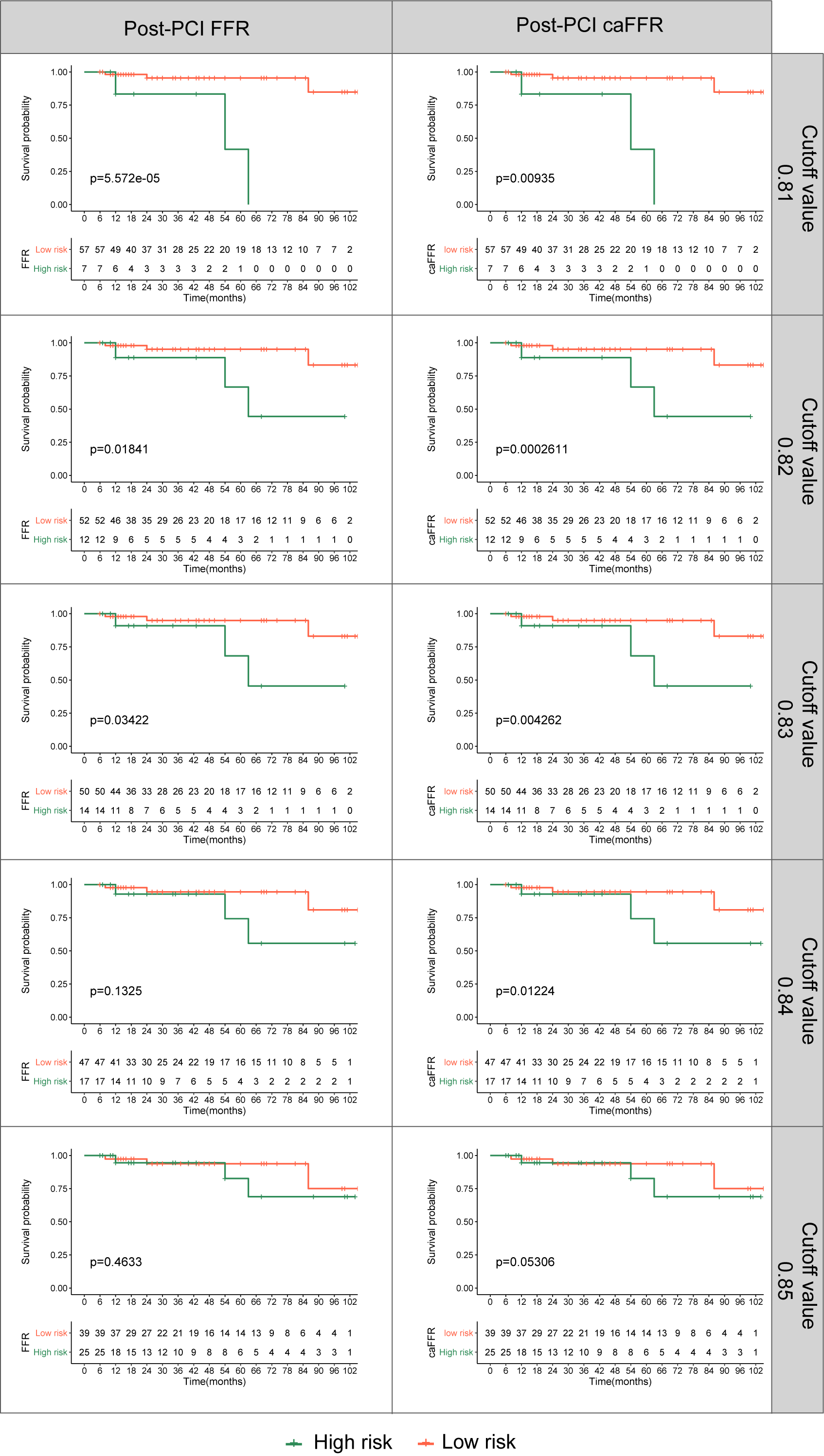

Supplement: Supplementary Figure 1 — Subjects whose post-PCI wire-based FFR or caFFR values were lower than the cutoff values were categorized as the high-risk groups. LAD, left descending artery; FFR, fractional flow reserve; PCI, percutaneous coronary intervention; caFFR, coronary angiography-derived FFR. [file Image_1.TIF]
